# Computational Creativity and Music Generation Systems: An Introduction to the State of the Art

**DOI:** 10.3389/frai.2020.00014

**Published:** 2020-04-03

**Authors:** Filippo Carnovalini, Antonio Rodà

**Affiliations:** Department of Information Engineering, CSC - Centro di Sonologia Computazionale, University of Padova, Padua, Italy

**Keywords:** computational creativity, music generation, survey, meta-review, algorithmic composition, musical metacreation, automatic composition, computer music

## Abstract

Computational Creativity is a multidisciplinary field that tries to obtain creative behaviors from computers. One of its most prolific subfields is that of Music Generation (also called Algorithmic Composition or Musical Metacreation), that uses computational means to compose music. Due to the multidisciplinary nature of this research field, it is sometimes hard to define precise goals and to keep track of what problems can be considered solved by state-of-the-art systems and what instead needs further developments. With this survey, we try to give a complete introduction to those who wish to explore Computational Creativity and Music Generation. To do so, we first give a picture of the research on the definition and the evaluation of creativity, both human and computational, needed to understand how computational means can be used to obtain creative behaviors and its importance within Artificial Intelligence studies. We then review the state of the art of Music Generation Systems, by citing examples for all the main approaches to music generation, and by listing the open challenges that were identified by previous reviews on the subject. For each of these challenges, we cite works that have proposed solutions, describing what still needs to be done and some possible directions for further research.

## 1. Introduction

What is *Creativity*?

While the term is of fairly common use in everyday life, giving a precise definition of this concept is not a trivial task. The general idea is that it relates to the ability that some human individuals possess to create something that did not exist before. Upon further reflection, one can notice that most of the times these “creations” start from concepts that already existed, or at least that could already have existed, but that nobody had already explicitly linked in a fixed product. This kind of “novel linkage” is what brought us works of art such as Dals The Persistence of Memory: clocks had been painted before, and everybody has experienced that things can melt, but nobody had yet linked these two concepts in a painting.

There is another question relating to creativity that raises even more problematic considerations: can computers be creative? The usual experience with machines is that we humans give a set of instructions to the machine along with some initial data (the input), and we expect the machine to behave in a way that is fully deterministic, always giving the same output when the same input is given. Moreover, we expect that the output should be something that can be fully expected and computed even without the help of a computer, albeit the computation of the output could be extremely time-consuming (otherwise we would not have resorted to computers in the first place).

The word deterministic seems to be the exact opposite of our understanding of the concept of creativity, and yet the idea of obtaining creative behaviors from computers has inspired the writing of a notable amount of scientific publications, that can be collected under the field of *Computational Creativity* (CC), defined as:

“The philosophy, science and engineering of computational systems which, by taking on particular responsibilities, exhibit behaviors that unbiased observers would deem to be creative.” Colton and Wiggins (2012).

Practitioners of this field share the interest in gaining a better understanding of how creativity works, and to what extent it can be replicated via a computer system. The above definition underlines the diversity in background of the people researching CC. Such a diverse community is for sure source of many interesting insights, but there is also room for many different goals and perspectives that sometimes can make it hard to understand what is the current general direction of the research, and what directions should be explored for the advancement of the field (Lamb et al., 2018).

One of this field's goals is for sure answering to the above question can computers be creative?, that is most interesting to computer scientists and engineers that wish to create advanced models of artificial intelligence with creative capabilities. Yet, this is only one of the possible goals: artists could be more interested in finding out how computers can help express their own creativity, while psychologists and philosophers are more interested in using computer models of creativity to better understand creative processes that happen in humans (Pearce et al., 2002). The diversity of the goals is reflected in the diversity of the literature concerning CC. Some works are devoted to the definition or the assessment of creativity itself. A notable amount of contributions focus on the design of systems that are meant to be creative, sometimes designing them starting from some definition of creativity, and thus trying to actually obtain output that is creative according to a certain evaluation method. Other times, the system is simply designed to tackle tasks that are commonly considered to involve creativity (usually artistic tasks like painting or composing music), but the process involved in the generation of such output does not necessarily involve creativity. More often than not, the creativity of the creative systems is not evaluated in any formal way, either having just summary evaluations of the quality of the output or not having any evaluation at all (Jordanous, 2012). This might be fine for the artistic goal of empowering the creativity of humans through computational means, but is not acceptable for AI practitioners trying to understanding whether computers can exhibit creative behaviors.

One especially prolific research task of CC is that of music generation, that has interested computer scientists even before the birth of the term Computational Creativity. Ever since the early days of computing, scientists and engineers have used computers for musical task, creating digital synthesizers, developing engraving software, and also writing procedures that generate musical scores, to be performed either by computers or by humans. This task was called Algorithmic Composition, and as the name suggests was related to a well defined procedure (an algorithm, once again a concept distant from creativity Papadopoulos and Wiggins, 1999; Nierhaus, 2009; Fernández and Vico, 2013. A name that is more common today is Musical Metacreation, that suggests the fact that the programmer creates a system that in turn can create some kind of music Pasquier et al., 2017; Bodily and Ventura, 2018. Throughout this article, we will use the more neutral term “Music Generation Systems” (MGSs). Music is especially interesting for the investigation in CC because of the broad possibilities that it offers in terms of mathematical and computational representations, and because it does not need explicit semantics like other forms of art such as poetry or non-abstract painting (Wiggins, 2018). This might be some of the reasons why there is so much research on MGSs, and also many reviews of the literature. Those reviews usually focus on the technical approaches used for music generation rather than the contributions given to the understanding of creativity in computers and humans. What this survey tries to accomplish is to give a broad introduction to the field of CC with a focus on MGSs, first reviewing the literature on the definition and evaluation of creativity and then focusing on the systems proposed in literature, describing the current approaches and the challenges that are still not fully addressed, and what kind of solutions have been tried or proposed to overcome those problems.

## 2. Formalizing Creativity

### 2.1. Defining Creativity

We opened the introduction to this paper asking what is creativity. This is a question that many researchers have faced before, especially after Guilford's speech to the American Psychological Association in 1950 advocating for psychological studies on creativity (Rhodes, 1961). Since that year, psychological research on creativity exploded, exploring many facets of what defines and stimulates creativity in humans. Some works were focused on the study of what are the personality traits of the creative person (Rhodes, 1961; Getzels and Jackson, 1962), as well as what external factors can positively or negatively influence creativity (Amabile, 1983a,b). Other researchers were more interested on the mental processes that happen in the creation of something creative, and finally many works were dedicated to the definition of creativity itself.

#### 2.1.1. Creativity as Novelty and Value

The newfound abundance of research led to having hundreds of definitions of creativity in literature. In their works, Sarkar and Chakrabarti analyzed over 200 of those (Sarkar and Chakrabarti, 2008, 2011; Ranjan et al., 2018), finding that the factors that have been used as indicators for creativity can be grouped in two main categories: *Novelty* (or unusualness, unexpectedness, surprise, originality) and *Value* (or usefulness, quality, appropriateness, meaningfulness). This subdivision is not new at all, as already Stein (1953) had proposed a definition of a creative work as novel and useful or satisfying. Novelty is usually considered the defining characteristic of a creative artifact, but value is also necessary: it is easy to think of something that has never been built before, like a car with fifteen wheels, but while such car would be novel, it would have higher maintenance costs, with little or no increase in performance. This kind of novelty lacks value: creativity (that includes value) introduces innovations useful to the purpose of the created object, possibly leading to a general advancement in its own field. One example of creativity in the field of car manufacturing could be the introduction of hybrid cars: the idea of using two different energy sources was novel, but hybrid cars are now common because of the advantages they bring to their owners in terms of efficiency. On the contrary, it is highly unlikely that our fifteen-wheeled car could become an industrial standard.

While Novelty and Values are surely important features of creativity, these give only a vague description of creativity. From their study of the literature, Sarkar and Chakrabarti (2008) reached a somewhat more complete definition of creativity:

“Creativity occurs through a process by which an agent uses its ability to generate ideas, solutions or products that are novel and valuable.”

#### 2.1.2. The Four Perspectives of Creativity

The above definition points out that creativity is a concept that cannot be ascribed to the final artifact, but must consider its creation. In particular, it underlines the existence of a process, used by an agent, to create a product. These represent three of the four “P's” of creativity, first identified by Rhodes (1961): Person, Process, Product, Press. Rhodes was interested in the educational aspects of creativity and in educating children to be creative, but later the focus shifted toward the study of what makes something be considered creative. This shift is probably partly due to a paper by Anna Jordanous, who revisited the concept of the four P's under the light of the evaluation of creativity (Jordanous, 2016), but the definition of the four P's had already started changing soon after the original paper by Rhodes (Golann, 1963), showing that while the general idea of the four P's was immediately utilized by the scientific community, it took some time to reach widely accepted definitions. Lamb et al. (2018) describe the four terms as follows:

- **Person**: is the human (or non-human agent) who is seen as creative. Person theories study what it is about the agent that makes them creative.
- **Process**: is the set of internal and external actions the agent takes when producing a creative artifact. Process theories study what sort of actions are undertaken when creative work is done.
- **Product**: is an artifact, such as an artwork or a mathematical theorem, which is seen as creative or as having been produced by creativity. Product theories study what it is about the product that makes it worthy of being called creative.
- **Press**: is the surrounding culture which influences people, processes, and products and which judges them as creative or uncreative. Press theories study what it is that leads a culture to view something as creative.

This useful subdivision into four perspectives helps frame the various contributions on creativity, as often each work focuses on only one or two of the above perspectives. For example, the definitions of creativity as Novelty and Value are focused on the Product, even if Sarkar and Chakrabarti's definition encompasses almost all four P's. In the following sections we review some contributions that focus on the other three perspectives: Person, Process and Press.

#### 2.1.3. Person

Regarding the Person perspective, the study of the personality traits of creative people has unsurprisingly interested many psychologists: already in the first years after Guilford's speech many works emerged (those early works were reviewed by Golann, 1963), and soon was found out that creativity is not directly related to intelligence (Getzels and Jackson, 1962), and a relationship between creativity and humor was also noted (Treadwell, 1970). Guilford himself underlined that creatives emerge for their sensitivity to problems, mental flexibility, and divergent thinking (Guilford, 1957, 1967). The importance of this last trait was exploited by Torrance, who designed the Tests of Creative Thinking (Torrance, 1965) that give an effective measure for the individuation of creative people (Torrance, 1988). Simonton (2000) gives a review of psychological studies on creativity in terms of personal and developmental traits, as well as the socio-cultural influence of creativity (connecting the Person and the Press perspectives).

Within the field of CC, one could argue that any Turing-complete machine is equivalent in what it can achieve, thus making every computer system equal under the Person perspective. Nonetheless, the Person remains an insightful perspective at a more abstract level, for example when a software system can be viewed as an agent or as a group of agents collaborating together. In this case, the (virtual) personality of each agent could give a different contribution to the system, making it useful to consider psychological personality aspects such as motivation (Guckelsberger et al., 2017) or curiosity (Schmidhuber, 2012), or to try and model in software cognitive aspects of creativity (Wiggins and Forth, 2015; Wiggins and Sanjekdar, 2019).

#### 2.1.4. Process

The Process perspective has interested CC the most, as someone who wishes to obtain a creative behavior from a computer must know how to describe creativity in algorithmic terms. While there is no such thing as a fixed procedure to obtain something creative, it is possible to gain insights on how to obtain creativity from the study of the creative processes of people that have shown great creativity throughout history (and wrote how they reached that idea). This is in part what Margaret Boden did in her book, The Creative Mind (Boden, 2004) (for a shorter introduction to the same ideas, see Boden, 1998, 2009). The description of creativity she provides in that book has become extremely influential to the field of CC, also because she used computer models of creativity to discuss her ideas, explaining what was obtained and what was still to be achieved by machines. One major contributions she gave was the introduction of the idea of “Conceptual Space,” i.e., a space where the possible concepts exist, some of which have been explored and some are yet to be discovered. This idea allows the distinction of many levels of creativity:

- **Combinational Creativity**: two already explored ideas from a concept space are joined, thus creating an association that is novel;
- **Exploratory Creativity**: some kind of method for the free exploration of the concept space is used, to find regions in the space that have not been yet explored, but are valuable;
- **Transformational Creativity**: the highest level of creativity is reached when a new idea is found that was not part of the original conceptual space, thus changing the shape of the concept space itself.

The idea of obtaining creative ideas from the union of two known ideas, that Boden called Combinational Creativity, is at the basis of other theories of creativity, although with different names: Koestler (1964) called the same idea *Bisociation*, while Fauconnier and Turner (2008) used the term *Conceptual Blending*. The novelty of Boden's theory lies in the introduction of conceputal spaces, necessary for the definition of the other two levels of creativity. Wiggins (2006, 2019) mathematically formalized these ideas, also showing that Transformational Creativity is equal to Exploratory Creativity on a meta-level.

Another useful notion introduced by Boden is the distinction between *H-Creativity* (historical creativity) and *P-Creativity* (personal creativity). In order for something to be H-Creative, it must be the first time it has appeared in the history of mankind, while to be P-Creative it is enough to be new to the one creating it. As an example, Boden mentions that if a child can prove Pythagoras' theorem without any help, we would find this deed an impressive example of mathematical creativity even if that theorem was demonstrated millennia ago. H-Creativity is what is usually considered novel and/or creative, but Boden argues that P-Creativity is just as important as it originates from the same creative Process.

#### 2.1.5. Press

The Press perspective is most interesting to the evaluation and assessment of creativity. This is not just an appendix to the concept of creativity: the definition we gave for CC seeks behaviors that are deemed to be creative by an unbiased observer, making it necessary to have an external appraisal of the Product before calling something creative. The works of Amabile have underlined both the importance of the environment for the development of creativity (Amabile, 1983b; Amabile et al., 1996) and the importance of the assessment of creativity, proposing one of the first formalized methods for the evaluation of creativity, using expert judges (Amabile, 1983a). Moreover, Csikszentmihalyi (2013) pointed out the proactive function that field's experts can have in increasing the rate of creativity in a particular domain. We will discuss the problems relating to the evaluation of creativity later (see section 2.3).

Even if someone tried to directly assess the creativity of a Product, of the Process behind it, or of the Person, he needs to pass through the lens of human perception (and thus the Press perspective) to be really understood (Colton, 2008), making the Press perspective the most ubiquitous. On the other hand, the Press perspective is not enough to give an indication of creativity, since commercial success or reach of a Product is influenced by a variety of factors that go beyond creativity, or even just its Value (Fraiberger et al., 2018).

#### 2.1.6. Dimensions of Creativity

Another interesting contribution to the definition of Creativity comes from Jordanous and Keller (Jordanous, 2012, 2013, 2019; Jordanous and Keller, 2012, 2016), who used a statistical language processing techniques to identify fourteen main components of creativity, as described by scientific research on the topic. This study resulted in an unordered list of components, that should be seen as different dimensions of the concept of creativity rather than a systematic description (Jordanous and Keller, 2016):

- Active Involvement and Persistence;
- Dealing with Uncertainty;
- Domain Competence;
- General Intellectual Ability;
- Generation of Results;
- Independence and Freedom;
- Intention and Emotional Involvement;
- Originality;
- Progression and Development;
- Social Interaction and Communication;
- Spontaneity/Subconscious Processing;
- Thinking and Evaluation;
- Value;
- Variety, Divergence and Experimentation.

The notion of Novelty (here called Originality) and Value are kept, but using all 14 components gives a much broader definition of creativity, that considers all the four Ps: for example *General Intellectual Ability* is related to the Person, *Progression and Development* to the Process, *Value* to the Product, and *Social Interaction and Communication* is connected to the Press perspective. Jordanous and Keller (2012) explain that not all the components listed above will be as important in all possible creative deeds, so this list also offers the possibility to categorize different kinds of creativity required by different activities.

To our knowledge, there is no work in literature that has given a short definition or a model of creativity based on these fourteen dimensions.

### 2.2. Computers and Creativity

The above definitions of creativity were general enough to be applied both to humans and machines alike (although we sometimes focused on the implication of those theories on computers). It is now time to face the second question we posed in the introduction: can computers be creative?

This is a question that seems to be as old as computer science: Lady Lovelace, while commenting the Analytical Engine, mentioned that computers do not have the ability to originate anything on their own (Lovelace, 1843). As paraphrased by Bringsjord et al. (2003), her statement reads:

“Computers can't create anything. For creation requires, minimally, *originating* something. But computers originate nothing; they merely do that which we order them, via programs, to do.”

The Countess leaves no room whatsoever for creativity, but other important scientists disagreed with her. Alan Turing, who argued that artificial intelligence should have creative abilities, responded to Lady Lovelace's objection pointing out that she had no real experience in programming, while we now know that a computer can often surprise us by doing the exact opposite of what we intended, until a program is thoroughly checked for bugs (Turing, 1950). This response is somewhat unsatisfying, since it seems that the only accountability for creativity from computers would come from human errors, but in the rest of the article Turing argues that intelligent machines should be able to learn, thus gaining abilities beyond those envisioned by the original programmer.

Another strong argument against computer creativity is that of the “Chinese Room” introduced by Searle (1980). He argues against artificial intelligence in general, but the argument applies to creativity as well. He imagines to be locked inside a closed room, that can accept questions and give answers written on paper, either in English or in Chinese. For the English questions, he would answer normally using his own intelligence, while for the Chinese ones he would use a special script telling him, for any combination of Chinese symbols that he sees, what symbols to write as answer. Supposedly, the English answers would be as good as the Chinese ones to the eyes of the people outside the room (if the Chinese script is good enough), but the person inside would not gain any knowledge of Chinese in this way. Searle argues that computers work in this way, manipulating symbols without having a real understanding of those.

Searle's objection is rather convincing, unless we suppose that the manipulations of symbols that happen in computers are in reality not different from those that happen in our brains, if not because of less “computational power” (Minsky, 1982). This vision basically reduces human brains to extremely powerful computers, so that an artificial computer could recreate all of their functions. This is of course far from being a proven truth, and does not fully account for things we experience everyday, such as consciousness, free will, and subjectivity (Chalmers, 1995; Hameroff and Penrose, 2014; Ceroni and Prosperi, 2018).

There is room for a long lasting debate on the possibility of computers being “*really*” creative, but fortunately CC is not ultimately interested in this debate. According to the definition of CC, we want computer systems that have behaviors that an unbiased observer would deem to be creative, and not necessarily behaviors that are *actually* creative. This means that we aim at simulating creativity well enough to trick observers into thinking that the product they are seeing is actually creative.

It is nonetheless important to understand what creativity is, and possibly to incorporate the definitions of creativity in the generation process, because the unbiased observer will judge creativity in the same way as it would with a human, thus implicitly applying some of the concepts relating to creativity that we illustrated above. The problem of the evaluation of creativity thus becomes central: if the goal is to recreate what an observer would deem creative, we need to give metrics of how creative something would be perceived by an observer.

### 2.3. Evaluating Creativity

Despite the importance of the evaluation of creativity, most of the scientific publications on evaluation only came about in the last 20 years (Jordanous, 2013). In this section we will describe some of the most common creativity evaluation methods. To read some more extensive reviews on this subject, we suggest: Jordanous (2012, 2013, 2014), Lamb et al. (2018), Pease and Corneli (2018), and Ritchie (2019).

#### 2.3.1. Turing Test-Like Approaches

The definition of CC that we gave suggests that creativity needs to be assessed via human judgement, leading to evaluation techniques based on the concept of “Turing Test” (Turing, 1950): ideally, if a human cannot distinguish computer creativity from human creativity, the computer has achieved a satisfying level of creativity.

Amabile (1983a) proposed the Consensual Assessment Technique, which has become the standard evaluation of human creativity (Baer and McKool, 2009). This technique requires a pool of experts independently evaluating a set of artifacts. An artifact can be considered creative if it receives good evaluations and the interrater reliability is high enough (for example having a Cronbach's alpha higher than 0.7). While this method was not originally conceived for CC, it is easy to insert one or more computer generated artifacts along some human made ones, to get a comparison between human and computer creativity. The judges only have access to the artifact, not knowing anything about its author or background (including whether the author is a computer). This means that they only evaluate the Product perspective in a non interactive way. This is rather different from the original Turing Test, but it was included in this section because it operates a comparison between human and computers carried out by a human evaluator.

Pearce and Wiggins (2001) propose a machine composition framework that includes in its final phase an evaluation inspired by the Turing Test (although the authors underline the major difference of not having interaction). While it was initially defined for music generation, it can be applied to CC in general. This framework supposes that a corpus is available to the software, and that some sort of learning is applied to create a “critic” for that corpus. Once new compositions are generated that satisfy the learnt critic, some generated pieces are presented a group of subjects along with composition coming from the corpus. The evaluators are asked to tell whether the compositions they hear are human or machine made (similarly to Turing's imitation game). If their evaluation cannot be statistically distinguished from a random selection, the system is considered effective. This approach, being entirely based on learning a corpus, is arguably not really an evaluation of creativity but rather one of quality in imitating human products.

Ariza (2009) underlines this and other limitations of Turing Test approaches to the evaluation of creativity, showing how sometimes these tests are implemented in a way that he calls “toy Tests,” failing to understand that interactivity between human and computers was the main feature of the “Imitation Game,” as it was meant to assess intelligence, that is experienced through interaction. Another critic to this kind of tests comes from Soldier (2002), who raises a more fundamental doubt on the capability of non-experts to act as evaluators. This is not surprising (indeed, the Consensual Assessment Technique requires experts), but often Turing Test approaches only require the evaluator to be human.

Bringsjord et al. (2003) propose to go beyond the Turing Test with the “Lovelace Test” (inspired by her statement reported in section 2.2). The authors argue that Turing's game could be beat with simple manipulation of symbols without the need of any intelligence (as Searle described with his Chinese Room example). On the contrary, an agent passes the Lovelace Test if and only if it is capable of creating an output of some kind through a repeatable process, and this output cannot be fully explained by the knowledge-base, the architecture, and the core functionalities of the agent. Unluckily, this test is not easy to perform in real-life situations, and arguably a machine could never pass this test, as every output of a machine is the result of its architecture and functionalities. This might be a good abstract test for *real* creativity, but is not very useful to evaluate CC systems.

A more manageable version of the Lovelace Test was proposed by Riedl (2014), that requires the machine to be able to generate an output that satisfies a set of requirements chosen by a human. The generated output is then evaluated in terms of how well it meets the requirements and if it is “not unrealistic for an average human.” This proposal is somewhat unsatisfying, because by losing the strong requirements of the original Lovelace Test it basically falls back to a standard Turing Test, in a way that Ariza (2009) described as “Directive toy Test,” meaning a Turing Test where the interaction is only limited to giving initial directives for the generation.

#### 2.3.2. Self-Assessment Frameworks

Another popular approach is to have the author of the system describe the way it works and how it can be considered creative or not, and to what degree. These assessments try to frame the chosen Process in some kind of creativity scale, for example distinguishing if the used process is combinational, explorational or transformative, using Boden's categories. Indeed, this kind of evaluation is reminiscent of how Boden investigated creativity in her book (Boden, 2004).

Colton (2008) introduced these assessments with a reflection on how the evaluation of the Product alone is not enough to evaluate the creativity of a system. He proposes an example, where the same object is obtained through different processes. This can lead to different perceptions of creativity, but obviously only if the process is known to the observer. In that paper, he introduced the concept of the “Creative Tripod,” a tripod having *Skill, Appreciation* and *Imagination* as legs, saying that all three must be extended to some degree in order for the tripod to stand.

The tripod framework had little success, possibly because it was not formalized enough, but it remained influential on literature on creativity evaluation. Colton et al. (2011) and Pease and Colton (2011a,b) described another framework for self- assessment: the FACE and IDEA models. The FACE model can be used to describe the creative capabilities of a system through a set of symbols that tell if the evaluated system *possesses* or is *capable of generating* Expressions (i.e., products), Concepts, Aesthetic measurements, and Framing information (read backwards, the initials spell FACE). The IDEA model describes instead the impact of the system during its lifecycle, starting from the developmental stage and ideally reaching a stage where it can perform some kind of transformational creative processes.

These assessment frameworks are limited in the possibilities they offer, and a common criticism is that the assessment comes from the author of the system, making it biased. Nonetheless it is useful to frame the capabilities of a system and to reflect on the degree of automation in creativity it has reached, even just for development purposes. Indeed, an extension to the FACE/IDEA framework was proposed to consider the creative abilities of different versions of a same software (Colton et al., 2014) to make it easier for a developer to understand how the creativity of the system is progressing.

#### 2.3.3. Quantitative Metrics

In order to compare the results of different systems in terms of creativity, and to give more scientific indications of the effectiveness of CC applications, it is desirable to have objective metrics that can indicate how creative a system is. Designing such metrics is not an easy task, but many efforts have been made toward this goal.

Ritchie (2001) proposed a set of criteria for the evaluation of creativity based on the Product perspective, judged according to *Value* and *Typicality*. The latter is a concept strongly related to Novelty, but is based on the fact that an “inspiration set” (the corpus used by the system) is available, and used to define what is more or less typical. These two basic features must be measured according to some *rating scheme*, and can then be used to compute a set of parametrized criteria, that are basically functions over the Value and Typicality. In his proposal Ritchie described these criteria as either satisfied or not satisfied (if a certain threshold is reached), but often these were applied as a continous scale rather than a boolean one. Extending this evaluation framework (Pease et al., 2001) focused on the measurement of Value and Typicality, while Colton et al. (2001) investigated the effects of fine-tuning the input knowledge. Ritchie (2007) presented an updated version of his criteria, commenting the works that have used it as a means of evaluation, but the presence of many parameters to be tuned makes it difficult to use for comparisons between different systems.

While Ritchie's criteria are the main metrics for the evaluation of creativity, there are other metrics in literature that can be relevant for CC systems, although they do not evaluate directly the creativity of the systems. Galanter (2012) made a review on metrics and methods to evaluate aesthetic value of computer generated artifacts, that is a vital part of many CC systems. Within the field of computer vision, the use of fuzzy logic applied to visual features was suggested for the automatic evaluation of complexity, as well as interestingness and aesthetic value (Cardaci et al., 2009; Tabacchi and Termini, 2011; Constantin et al., 2019). Shaker et al. (2016) focus on procedural content generation, and describe how it is possible to give a visual indication of the capabilities of a system in terms of the variety of products it can generate. To the best of our knowledge, this system has never been used for CC systems, despite the fact that the representation of the space of possible outputs generated by a system has strong links to Boden's theories (which in part inspired Shaker's work). Possibly, these graphical representations could give a good indication of whether a system uses mere combinational creativity or is capable of going beyond that limit.

#### 2.3.4. Evaluation of Generated Music

The evaluation methods that we presented in the previous paragraphs are general enough to be applied to musical generation as well as to other CC applications. The following methods focus instead solely on the evaluation of MGSs.

Eigenfeldt et al. (2012) used a concert setting to evaluate a variety of MGSs, and a similar event is described by Sturm et al. (2019). In both cases, the evaluation in itself was performed via a questionnaire given to the audience of the concert. This approach can be extended by turning the concerts into music competitions, as has been done for computer-generated expressive performances of human composed music (Katayose et al., 2012; Schubert et al., 2017). If the program includes both human and computer generated music, this approach becomes similar to the ones inspired by the Turing Test, but a concert setting is one of the most natural ways to experience music, and could fatigue the evaluators less than a laboratory setting. Two major limitations of this approach is that the audience will evaluate music according to their personal taste, rather than assessing creativity, and that this evaluation method can only be used to compare the pieces that are included in the concert: comparing different concerts could induce unwanted bias due to different performers, venue and setting in general.

Another useful contribution is that of Yang and Lerch (2018), that argue that while creativity cannot be assessed without a human evaluator, it is useful to use *formative* metrics to describe how well computer generated music fits a musical genre, in order to help the development of the system toward “human-like” music generation. To that goal, many quantitative metrics are presented, and data visualization techniques are suggested. While this does not solve the ultimate goal of the evaluation of creativity, it is nonetheless an useful addendum to the evaluation toolbox.

An overview of the current methods for the evaluation of MGSs is present in Agres et al. (2016), that provides both motivations and tools to evaluate in different manners systems that are merely generative, systems that allow for feedback, and systems that are capable of some kind of self-reflection. Moreover, a distinction is presented between internal and external evaluation, the first being necessary for the functioning of the system and the latter being the usual *a posteriori* evaluation to understand the effectiveness of the system.

## 3. Music Generation Systems

### 3.1. Meta-Review

This is not the first review on Music Generation Systems (MGSs), and the goal of this work is not to give a comprehensive review of every contribution to the field, but rather an introduction through examples from literature. To this goal, we searched on Scopus and Google Scholar for reviews on MGSs that have been published over the last 10 years (2009–2019), by searching “computational creativity music,” “musical metacreation,” “algorithmic composition,” and “music generation” followed by “review” or “survey,” limiting to first 50 results. From the results, only the papers written in English after 2009 were kept. Of those, the abstract was read to select those that were actually reviews of artificial intelligence techniques for music generation. The selected ones are listed in Table 1. Two results (Williams et al., 2013; Briot et al., 2017) were excluded because they were prior versions of the reviews we included by the same authors.

**Table 1:** Summary of the reviews used as a starting point for the present survey.

	**Focus**	**Taxonomy**	**References**	**Mentions to creativity**	**Pages**
Nierhaus (2009)	Broad review of all the methods for algorithmic composition in literature.	Method	328	24	294
Fernández and Vico (2013)	Broad (yet condensed) review of all the methods for algorithmic composition in literature.	Method	337	38	70
Williams et al. (2015)	Affective/emotional assessment integrated in algorithmic composition.	Expressive features	123	0	24
Herremans et al. (2017)	An objective-based taxonomy to better understand the state of the art in music generation.	Objective and method	165	4	30
Lopez-Rincon et al. (2018)	Brief introduction on a variety of AI methods used for music generation.	Method	32	2	7
Tatar and Pasquier (2019)	Generative musical agents.	Typology of agents	205	122	50
Briot et al. (2020)	Music generation using deep learning techniques.	Method	212	37	303

*For each, the focus for the choice of works to be reviewed is reported, as well as what the classification of the systems is based upon (Taxonomy), the number of references present in the reviews, the number of times creativity is mentioned, and finally the page count*.

It is important to notice that not all the works on MGSs have the goal of CC in mind. Sometimes the goal of a MGS is to create a formalization of a certain musical style, or to test certain composition rules or assumptions by generating music that satisfies those rules. Other times, the generation of music itself is the only goal of those systems. This is also reflected by the reviews on MGSs, that are not always concerned with the creativity of the reviewed systems: we used search tools to count the number of occurrences of the stem “creativ” in the main body of the reviews, that we reported in the Table 1 under the column “Mentions to Creativity” to show that some reviews on music generation hardly acknowledge the problem of creativity at all. The goal of the reviews varies as well. Older reviews made comprehensive lists of methods for MGSs, while newer reviews tend to focus on more specific subsets of the literature. A brief description of the aim of the included reviews is listed under “Focus.” Since every review tends to group works in clusters, we listed the criterion for the subdivision of the reviewed works under the column “Taxonomy.” Finally, for each review we included the number of pages and the amount of references in their bibliography.

Some reviews also include sections or chapters are not directly related to MGSs: Nierhaus (2009) includes a chapter narrating the history of Algorithmic Composition; Williams et al. (2015) gives a brief review of studies that investigate emotional correlates of musical features; Herremans et al. (2017) gives an introduction both to the history of Algorithmic Composition and to the problem of Evaluation that we discussed in section 2.3. Finally, Briot et al. (2020), gives an introduction both to the ways in which musical data can be encoded and to deep learning in general. Moreover, to exemplify some deep learning techniques that were not yet used in MGSs, the authors cited some *visual* generation systems.

### 3.2. Methods for Music Generation

Many algorithms and techniques were applied to music generation, but it is possible to group those in some main categories. The subdivision we use is the one used by Fernández and Vico (2013), which is in turn based on prior reviews (Papadopoulos and Wiggins, 1999; Nierhaus, 2009). More recent reviews have either used this taxonomy or expanded specific subsets of its six classes. We decided to add a seventh category for Agents based systems, which is a meta-approach that has gained a lot of popularity and deserves to be treated separately. The seven categories are:

- Markov Chains;
- Formal Grammars;
- Rule/Constraint based systems;
- Neural Networks/Deep Learning;
- Evolutionary/Genetic algorithms;
- Chaos/Self Similarity;
- Agents based systems.

In the following sections, we will describe each of these approaches by citing works that implemented MGSs using techniques that fall in those categories, also briefly discussing how these approaches can be seen under the Process perspective using Boden's categories of creativity (see section 2.1.4).

#### 3.2.1. Markov Chains

A Markov chain is a special stochastic process, i.e., a sequence of random events dependant on a time variable, that has a finite number of states, and the probability of the next state is only dependant on the current state (Brèmaud, 2013). In practice, a Markov chain is described by a transition table, where rows and columns represent the states, and every cell (*x, y*) represents the probability of going from the state *x* to the state *y*. Since each row represents a probability distribution, the sum of all the cells in a row must be equal to 1.

If the last *n* states are used to determine the probability of the next state instead of just the last one, this is called *n*-th order Markov chain. These can be represented with a single transition matrix as well, by constructing an equivalent first order Markov chain having *A*^*n*^ rows, where *A* is the number of states in the *n*-th order chain.

Due to their sequential nature, Markov chains are well fit to describe melodies, seen as a sequence of notes. The simplest way to implement a melody-generating Markov chain is to use a set of notes as the possible states, and to compute the transition probabilities between these notes by counting the occurrences of each transition in a given corpus to create a first order Markov chain.

This is what was done in one of the first MGSs ever described. Pinkerton (1956) created the “Banal Tune Maker” by analyzing the transitions of 39 nursery tunes by hand to create a transition matrix. The states used were the seven notes of the diatonic scale of C major (only one octave was considered), plus one extra symbol to indicate rests or notes that are prolonged over a beat. In this case the states of the chain only contain pitch information, requiring the use of other strategies to implement the rhythm. In this case, all the notes were kept to the same duration, and the extra symbol was used to introduce rests in the generated music. Of course, other approaches are possible, including implementing another Markov chain to handle durations.

The basic assumption underlying this simple approach, i.e., that the next note is only dependant on the previous note, is very flawed and only lead to musical results of little interest. Pachet (2002) used a more refined approach in the “Continuator.” He implemented a variable order Markov chains using prefix-trees to handle sequences of varying length (as opposed to *n*-th order Markov chains that will always consider *n* states) and also used a hierarchy of reductions: the system analyzed in a single chain pitch, duration and velocity, but was able to ignore some information when analyzing new input and comparing it to the learnt sequences. This was especially important in the Continuator because, as the name suggests, it was meant to listen to a musical input and continue it in real time. Being able to ignore part of the learnt information allowed the system to interact with previously unmet input, and to consider musical structures at various levels of detail.

Hiller and Isaacson (1958) used a different approach in their “Illiac Suite.” In their fourth experiment, they used Markov chains to generate sequences of motions and progressions rather than sequences of pitches and durations themselves, thus using the model to organize the notes at an higher level. The same idea of organizing higher structural levels via Markov chains was used more recently in the GEDMAS system (Anderson et al., 2013), whose goal was to generate Electronic Dance Music. To do so, a series of Markov chains were used to choose the general form of the song (i.e., a sequence of sections, each section being 8 bars long), to fill each section with a chord sequence, and finally to generate melodic patterns.

From the viewpoint of the creative process, Markov chains risk to reuse a lot of material from the learnt corpus non creatively, even plagiarizing when the order of the chain is too high (Papadopoulos et al., 2014). However, they can also result in novel combinations of smaller sections such as motifs, in a way that can be considered Combinational Creativity, but will hardly go beyond that limit unless other techniques are also employed. They also remain useful at higher structural levels (like the above example of GEDMAS), where high levels of creativity are not usually as important as when the melodic material is being generated.

#### 3.2.2. Formal Grammars

Chomsky (1957) introduced the concept of Generative Grammars, a tool for the analysis of natural language that became extremely influential in linguistic studies. The same idea was applied to musical studies, most notably by Lerdahl and Jackendoff (1985), who tried to design a Generative Grammar for the description of music starting from music analysis concepts introduced by Heinrich Schenker in his book “Free Composition” (Schenker, 1979), that well fit the concept of *rewriting rules*, that is at the basis of Chomsky's grammars.

A Generative Grammar is composed of two alphabets: terminal symbols and non-terminal symbols (or variables). A set of rewriting rules is given over the union of these two alphabets, that allow to transform variables into other symbols (both variables and terminals). The generated language is the set of all the strings of terminal symbols that can be obtained starting from a special variable chosen as starting point (usually called *S*) and applying any number of rewriting rules in sequence.

Grammars can be seen both as an analysis tool and as a generative tool. For example, Steedman (1984) compiled a Generative Grammar to describe Jazz chord sequences: Pachet (2000) describes a system that is in part inspired by Steedman's analysis to tell apart blues songs and non-blues songs, while Chemillier (2004) implemented Steedman's grammar creating a software for music generation.

Chord sequences can be very easily encoded as symbols, but, if an adequate alphabet is given, it is possible to use Grammars to generate any kind of musical information. Hamanaka et al. (2007) describe a system for the automatic analysis of scores based on Lerdhal and Jackendoff's Generative Theory of Tonal Music, formalizing in details a grammar to describe musical material. This was then used to create variations on melodies by altering the derivation trees (a graphical representation of the applied rewriting rules) (Hamanaka et al., 2008). Quick (2011) implemented a software to generate three voice harmonies using a Grammar derived from Schenkerian theory.

L-systems (Lindenmayer Systems) are a variant to Generative Grammars that has been used for music generation. Their main difference from Grammars is that they implement parallel rewriting, thus applying all the rewriting rules at once instead of only one at a time. This characteristic makes these system less apt to sequential data, like simple melodies, and have been used to generate stunning visual effects. When applied to music generation, the most common approach was to map visual data generated by L-systems either to score information (Prusinkiewicz, 1986; Mason and Saffle, 1994; Nelson, 1996) or to arrange a sequence of musical segments (Langston, 1989; Supper, 2001).

Formal grammars can be seen as a precise definition of a conceptual space, which is then explored when generating music. In this sense, the compilation of the rewriting rule can be seen as Transformational Creativity, but this is usually performed by a human rather than a computer. An exploration of the conceptual space of the possible rules can be seen as a meta-level creativity, which as Wiggins (2019) showed is indeed a form of Transformational Creativity, but this can be extremely hard to implement effectively in a CC system, since the compiling of a formal grammar requires careful study even when done by a human to ensure valuable results.

Another related approach is that of Transition Networks: finite state automata that can parse languages similarly to what Generative Grammars do. The most notable example of Transition Networks applied to MGS is that of David Cope's Experiments in Musical Intelligence (Cope, 1991, 1992). His approach was to use pattern-matching algorithms to analyze “signatures,” short musical sequences that define the style being analyzed, and to determine when and how to use those signatures. After the analysis phase, the collected information is encoded in a Transition Network that is then used to generate new music in the style of the composer that was analyzed. While the results are sometimes impressive, they are arguably not very creative, since they just reuse material taken from the learnt corpus in a way that can be at most be seen as Combinational Creativity (Wiggins, 2007). Possibly, this is one of the reasons why there is not much research on Transition Networks for music generation beside Cope's works.

#### 3.2.3. Rule/Constraint Based Systems

Music theory traditionally describes rules that help to guide the compositional process. While composers regularly break those rules, it should come to no surprise that those rules have been used to implement MGSs since the early days of Algorithmic Composition, like in the first two movements of the Illiac Suite (Hiller and Isaacson, 1958). Generative Grammars can be seen as an implementation of such rules, but the systems we refer to in this section are usually unable to generate musical material from scratch, and either start from some input material (like in the case of harmonization software) or use other methods, sometimes even random generation, to have a starting point that is then refined through rules.

The inclusion of rules can be implemented in many ways, for example as a final validation step, or to refine intermediate results. One natural way to implement rules in a MGSs is to use *Constraint Programming*, whose declarative nature is well fit to describe music theory rules. A survey on works that have used Constraint Programming to model music theory (not only with the goal of generation) can be found in Anders and Miranda (2011).

One of the most influential researchers within the scope of music generation through constraint is Ebcioǧlu, who first implemented rules of fifth-species counterpoint into a Lisp program, and later implemented a custom logic language that he used to create CHORAL, a system for the generation of Bach-like chorales that uses some 350 rules for the generation of melodies and harmonization (Ebcioǧlu, 1988, 1990). The difficulty of designing such a system lies in the complexity of explicitly coding a sufficient amount of rules, many of which often do not have a formal definition in musicology literature. Moreover, there is a tradeoff between adding more rules to obtain results that better fit the style that is being modeled and leaving less constraints to be more open to different styles of music.

Constraints can be used to model more abstract features, rather than explicit music theory rules: Herremans and Chew (2016b) defines a way to describe tension in musical pieces based on a geometric model of tonality called the *Spiral Array* (Chew, 2014). Herremans and Chew (2017) used that tension model in a MGS that is capable of generating new music following the tension pattern of an input piece, by first generating random notes and then applying optimization methods (in particular, Variable Neighborhood Search) to change the notes in order to satisfy constraints defined by the chosen tension model.

Techniques for optimization such as integer programming can be useful as a selection technique when more than one possibility is available. For example, Cunha et al. (2018) describe a MGS that creates guitar solos by concatenating guitar licks. This approach is somewhat similar to a transition network, but in their implementation the concatenation of any two licks had a defined transition cost, and through a branch-and-cut algorithm it was possible to compute the optimal solo. The computation of transition costs was in itself another example of integration of rules: in that work eight rules were described to assign the transition cost between licks.

The integration of rules and constraints in a creative Process can be see in two ways: the first is considering those rules as bounding and reshaping the conceptual space, the second is to see rules and constraints as a guidance in the exploration of the conceptual space. Either way, the use of rules can result in a more efficient Exploratory Creativity, although they might reduce the size of the conceptual space (or limit the explored areas) thus limiting the variety of the output.

#### 3.2.4. Neural Networks/Deep Learning

The increased computational power of computers and the widespread of general purpose GPU programming recently made deep learning techniques extremely popular, with applications that span from natural language processing, to image and video editing, to, of course, music generation. The survey by Briot et al. (2020) is specifically focused on these techniques, and gives an exhaustive overview of how machine learning has been used in MGSs.

While the interest in these algorithms grew exponentially in the last decade, the first MGS to use Artificial Neural Networks is that of Todd (1989), who used a three-layered Recurrent Neural Network (RNN) to generate monophonic melodies. Recurrent Networks reuse the results of the computations from previous steps when new input is given, allowing them to encode temporal sequences. This is of vital importance when generating melodies, making them a typical approach for MGSs that use deep learning (unlike, for example, Convolutional Neural Networks that are more apt for the elaboration of images). Nonetheless, there is also room for standard feed-forward networks: Lewis (1991) trained a network with musical patterns ranging from random to well-constructed, to learn a measure of “musicality” that is then used by his MGS to select pleasant compositions.

As already mentioned, RNNs are a popular choice for music generation. In particular, LSTMs (Long-Short Term Memory networks) Hochreiter and Schmidhuber (1997) are a special variant of recurrent networks that use special gates to decide the amount of information that is taken from novel input and what is maintained from older inputs. This control over the data flow allowed LSTMs to be both more efficient and effective than standard RNNs in a wide range of applications, and have been used for music generation as well. The first music generation LSTM was applied to blues improvisation (Eck and Schmidhuber, 2002a,b). Traditional music was instead the focus of *folk-rnn* (Sturm et al., 2016), that analyzed over 20000 pieces in textual (abc) notation. A more advanced approach is used by DeepBach, Hadjeres et al. (2017) that generates chorales in the style of Bach (whose chorales made the training set for the software) using two LSTMs, one going forward and one going backwards in time, together with one feed-forward network to consider contemporaneous notes. The results of these networks is then handled by a final feed-forward network that joins the results in final piece. The rationale behind this choice is explained by the goal of generating *counterpoint*, which requires knowledge of both the previous and the following notes. This gives an example of how it is possible to design complex architectures using many layers of Neural Networks, but the complexity comes with a price in terms of computational time.

Another deep learning approach that is of great interest to CC is that of Generative Adversarial Networks (GANs) (Goodfellow et al., 2014). The idea behind this method is to train two networks at the same time, one that generates artifacts imitating what is learnt from real-world examples, and the other trying to discriminate between real and imitated artifacts. As one gets better, the other must get better as well in order to “beat” the other network (thus making them “Adversarial”). The two networks can be simple feed-forward networks, but these are not the usual choice for music generation. For example, the eloquently called C-RNN-GAN (Mogren, 2016) uses recurrent networks (in particular LSTMs) in an adversarial architecture to generate polyphonic music. MidiNet (Yang et al., 2017) uses convolutional layers instead: Convolutional Networks are trained to reduce the dimension of the input, usually starting from bidimensional input. This approach is often used on images, so when applied to MGSs the input of the Network is often some graphical representation of music, such as piano rolls. It is important to be aware that while images have two dimensions that are equivalent (both represent displacement in space), graphical representations of music show two non equivalent dimensions, usually pitch and time, possibly leading to less reasonable results (Briot and Pachet, 2018).

Machine learning has strong implications for what concerns creativity: Turing advocated that learning machines would have been the key to beating the Imitation Game (Turing, 1950), and the very concept of learning is strongly related to the possibility of expanding and changing both the conceptual space and the means of exploring it, possibly reaching Transformational Creativity. However, Boden (2004) argues that connectionist systems cannot reach human levels of creativity, and Bringsjord et al. (2003) argues that the “learning” involved in a neural network is not enough to pass the “Lovelace Test” (see section 2.3.1). Both those works seem to only consider classic feed-forward neural networks rather than state-of-the-art deep learning approaches that are able to learn the representation and encoding of raw data (Briot et al., 2020), which can be seen as the definition of the conceptual space for these systems. From this viewpoint, these systems are the closest we have come to implementing Transformational Creativity, but the black box nature of these systems make it hard to pinpoint exactly how the generation process works and how the corpus information is used, making it also hard to control the output of the system (as we will discuss in section 4.1), a capability that is considered fundamental by certain definitions of creativity (Riedl, 2014; Jordanous and Keller, 2016).

#### 3.2.5. Genetic/Evolutionary Algorithms

The general idea behind Genetic (or Evolutionary) Algorithms is that, starting from a population of random solutions to a problem, it is possible to combine those solutions to obtain new solutions, and by selecting the ones that better answer the problem it is possible to get closer and closer to the optimal solution to the original problem. Thus, to solve a problem via Genetic Algorithms, it is necessary to have (Sivanandam and Deepa, 2008):

1. The ability to generate random but suitable solutions to the problem as a starting population;
2. A way to evaluate the “fitness” of a solution;
3. The ability to mutate and recombine those solutions.

In the field of music generation, the points 1 and 3 are for sure available (once a representation of musical material is chosen), but it is hard to evaluate how good a solution is (as already discussed in Section 2.3). It might be difficult even just giving a precise definition of what the problem is. Nonetheless, Genetic Algorithms have often been used to implement MGSs.

Possibly, the most famous Genetic MGS is GenJam, designed by Biles (1994). The system is meant for Jazz improvisation, where a human player interacts with the software that outputs both the pre-made musical base and solos generated on-the-fly by evolving the human improvisation it has just listened to. Originally, the fitness function was implemented by having a human decide if the output was good or bad, an approach that is usually referred to as “Interactive Genetic Algorithm.” This generates a bottleneck for the system, as a lot of human intervention is required. A successive version (Biles et al., 1996) used an Artificial Neural Network as a fitness function, but it lead to unsatisfactory results. In the end, the author resolved to completely eliminate the fitness function (Biles, 2001). Basically, the algorithm retains the ability to mutate and compose licks, an ability that is used to respond to musical input in a way that incorporates the human improvisation without being a mere copy, but since there is no more evaluation of the fitness, GenJam is no more a genetic algorithm.

GenJam passed, through his versions, some of the most common approaches to the definition of a fitness function. Another approach is to use rules taken from music theory to design a fitness function. This is the approach chosen by Phon-Amnuaisuk et al. (1999). In that case the goal was the harmonization of a given melody, and the fitness function incorporated rules of harmony describing forbidden and preferred intervals and motions. In this case, the use of genetic algorithms becomes a way to explore a space of possibilities described by the chosen rules. One might wonder if this is better or not than just generating samples following those rules, as described in the previous section. Indeed, Phon-Amnuaisuk and Wiggins (1999) found that their genetic implementation was outperformed by a rule-based system using the same set of rules that were incorporated in the fitness function. The authors argue that having explicit control over a system's knowledge will lead to better results and more powerful means of exploration: while the authors do not scorn genetic algorithms in general, it seems that this approach cannot give such explicit control over the knowledge of the system, and thus other systems should be preferred when explicit knowledge is available.

Genetic Algorithms offer many other forms of hybridization, since the representation used by other algorithms can be evolved genetically. For instance, it is possible to evolve the rules of a grammar (de la Puente et al., 2002), or to evolve the parameters of a Markov chain (Werner and Todd, 1997; Bell, 2011) or of a Cellular Automaton (Lo, 2012). We already mentioned that rules, Neural Networks and human assessments can be incorporated in the fitness function for a Genetic algorithm. It is worth mentioning that Markov chains have been used for the same goal (Lo and Lucas, 2006). Markov chains can also generate the initial population, obtaining starting point that is better than random, possibly leading to convergence to good solutions with fewer generations (Manaris et al., 2011).

The evolutionary approach is in itself an exploratory process: the combination of two individuals from the population pool is a combinational process, but the use of a fitness function guides the exploration toward promising areas of the conceptual space, which is bounded and defined by the genetic encoding of the individuals. Losing the fitness function, or having one that is unable to effectively guide the exploration, reverts the mechanism to pure combinational creativity, where elements of the conceptual space are joined and mutated hoping to find interesting unexplorated combinations.

#### 3.2.6. Chaos/Self Similarity

Musical compositions show some degree of self similarity, both in the musical structures and in its spectral density (Hsü and Hsü, 1991), roughly following a 1/*f* distribution, at least for pieces that are deemed pleasant to listen to (as opposed to random compositions) (Voss and Clarke, 1978).

Starting from these considerations, fractals and other self-similar systems have been used to generate musical material. The results of such systems are usually not regarded as a final output, but rather as an inspiration for human composers (Bidlack, 1992). Another approach is to generate self similar structures rather than directly generating self similar melodies: Leach and Fitch (1995) generated tree structures like those described by Lerdahl and Jackendoff (1985), by tracing the orbit of a chaotic system, and mapping the computed values to different hierarchical levels of the tree.

Another approach is to use Cellular Automata, dynamic systems composed of many cells, whose states are updated at discrete times using a set of transition rules. Famous examples include “Game of Life” by Conway (1970), and the systems studied in “A New Kind of Science” by Wolfram (2002)^1^. Like other fractal systems, Cellular Automata tend to generate melodies that are not too pleasing, and often need further human intervention. CAMUS is a MGS that is based on two different Cellular Automata, whose cells were mapped to sequences of notes and to different instruments (Miranda, 1993). A later version used a Markov chain to specify rhythm, but despite the effort to create a full MGS, the authors still admit that the results can often be not very pleasing, but can become interesting “for the composer who is prepared to put a little effort into the system” (McAlpine et al., 1999). Miranda (2007) later argued that Cellular Automata are more effective for sound synthesis, rather than for MGSs.

Since the decision making of these systems is based upon chaotic and random processes it is difficult to describe them using Boden's categories, and the usual lack of aeshtetic value of the results suggests that this is not a good example of CC but rather a way to explore unusual melodies. For these reasons, these systems are arguably less interesting to AI practitioners, but were included for completeness. Nierhaus (2009) provides a good review of these approaches, that are given less consideration by later surveys.

#### 3.2.7. Agents Based Systems

A software agent is an autonomous piece of software with perception and action capabilities. Any software with such capabilities can be seen as an agent (including many of the systems described in the previous sections), but the definition becomes especially interesting when multiple agents cooperate within a single software, that can be referred to as a Multi Agent System. This is not a specific algorithm for music generation, but rather a meta-technique that has gained popularity among researchers, as testified by Tatar and Pasquier (2019).

The use of agents in MGSs makes it easy to model certain musical behaviors. Voyager (Lewis, 2000) uses 64 player agents that generate melodies according to one of various pitch generation algorithms written by the author, according to his own taste, and a behavior model that describes the general timbre, tempo, pitch range and other features that regulate the development of the piece. This models a band where everybody is improvising, but still follows some general agreement. Lewis has played together with Voyager, both in recordings and live: in this setting one can also consider the human performer as one additional agent of the system.

MASs are also useful to model social interactions: once each agent is given specific characteristics (one could say, a personality), the interaction between different agents can take into account the difference in their characteristics, either in a conflict or in an agreement. For example, Kirke and Miranda (2011) introduces a system (later called MASC; Kirke and Miranda, 2015) where each agent has a specific “emotion” and the ability to express it by “singing” to another agent. The other agent will be affected by the mood expressed by the singer, adapting his own internal state. Moreover, their internal state also defines if the listener will “like” the song, incorporating it into his own song.

Taking further the same idea, the agents can implement cognitive models that regulates their interaction with the others. One such model is the Belief-Desire-Intention Architecture. For instance, Navarro et al. (2014, 2016) describe a system with the goal of generating harmonic sequences, where two particular agents, the *composer* and the *evaluator*, have beliefs based on music theory and desires (one to compose and the other to evaluate the generated composition). The intentions are represented by the algorithms implemented to apply and verify the theoretic rules that form their beliefs, and are influenced by the communication between the two roles.

Since the use of agents is a meta-technique rather than a specific algorithm, it is not possible to frame them from the Process perspective, but it is useful to consider the Person and Press perspective. The use computational means to give a “personality” is important to obtain results that are affectively relatable for humans, possibly making it easier to pass Turing-like tests. Moreover, the influence of other individuals is an important factor in human creativity (Amabile, 1983b), and is thus an interesting direction for research in CC (Saunders, 2019).

## 4. Open Challenges for Music Generation Systems

One of the goals of this review is to give pointers to any reader who is approaching Computational Creativity (CC) in general and Music Generation Systems (MGSs) in particular some pointers on what still needs to be addressed and the open challenges in the field. To do so, we extracted a list of problems and challenges that were identified in the reviews listed in Table 1, especially looking at those sections were the reviewers gave indications for future directions. The different surveys used variable terminology, often without giving precise definitions for the challenges they mentioned. To group them, we first tried to cluster those problems that we believed to be similar, and then gave precise definition for each cluster. We then re-read the problems descriptions in the reviews and marked as “mentioned” the clusters that was the closest to the descriptions. The clusters that were never mentioned were removed, and the remaining ones were given fitting names and are listed in Table 2 as the challenges we identified. The table also lists for each challenge which reviews mentioned it. The precise definition of each challenge is given in the next paragraphs. It is worth noticing that all the reviews mentioned Evaluation as an open challenge, and nearly half also mentioned Creativity (as opposed to mere imitation) as still lacking in most systems. Since we already widely discussed Evaluation, this won't be further treated. Creativity will be instead treated in each paragraph, as we want to make this review useful for CC as well as MGS research. To do so, we will try to categorize these challenges using the dimensions of creativity described by Jordanous and Keller (see section 2.1.6). For each of the other challenges, we will give precise formulations of what is the problem to be addressed, citing examples of works published in the last 10 years that have faced these problems and gave insights to what solutions could be used to overcome those problems and to achieve higher creativity.

**Table 2:** For each of the identified challenges, an X is added under every review that mentions it.

	**Nierhaus (2009)**	**Fernández and Vico (2013)**	**Williams et al. (2015)**	**Herremans et al. (2017)**	**Lopez-Rincon et al. (2018)**	**Tatar and Pasquier (2019)**	**Briot et al. (2020)**
Control							X
Narrative Adaptability				X		X	
Emotion			X	X			
Hybridization		X					X
Rendering	X			X			
Structure	X	X		X			X
Mapping	X						
Playing Difficulty				X			
Evaluation	X	X	X	X	X	X	X
Creativity	X	X					X

### 4.1. Control

*Control* refers to having the possibility to choose specific features that the output of the MGS will exhibit.

Having control over certain features of the output of a MGS can be, depending on the used algorithm, trivial. But, with more data-driven approaches like machine learning, it becomes less obvious what can be done to affect the output. It is not surprising that this issue was only mentioned in a review focused on deep learning.

Since data-driven approaches are meant to learn features from their input, one simple way to influence the features of the output is the selection of the training set. This approach is to some extent used by every corpus-based system, knowing that learning on Folk music will be very different from learning on Bach chorales. The problem with this approach is that it does not allow a good granularity of control, and any change on the input would require retraining the system, a task that can be very time consuming.

The same idea is applied in a slightly different fashion by Ekeus et al. (2012). Their approach was to generate a set of randomly sampled Markov chains, which were evaluated with an approach based on Information Theory. These were employed in a MGS that allows the users to select a point in a triangular space where the vertices represent periodicity, repetition, and noise. The chosen point is mapped to the features that were evaluated for each Markov chain, and the most appropriate one is selected and used for melody generation.

The same approach can be used in Neural Networks by altering the parameters that make up the network, but this can be much more intimidating, due to the excessive number of parameters involved and the difficulty of understanding their meaning (Sturm, 2018). A way to obtain this is proposed by Kaliakatsos-Papakostas et al. (2018), who used a recurrent network trained on a small dataset (made of only three pieces) that was augmented specifically to address the features the authors wanted the user to be able to manipulate, in order to study how the parameters are affected, and to be able to alter them accordingly in the generation phase.

Control is related to the creative dimension of “Active involvement & persistence” which suggests that the creative agent is in control of the generation process. Using deep learning to achieve this can be extremely hard, although many advancements in this direction are being made. We suggest to use techniques that allow for simpler tuning over the features one wishes to control, by either using appropriate representations (see section 4.5) or by explicitly limiting those features with rules. Machine learning can be used in conjunction with these approaches to ensure other creative features, such as “Variety, divergence & experimentation.”

### 4.2. Narrative Adaptability and Emotion

*Narrative Adaptability* refers to the capability of the MGS to convey a sense of development (Narrative) in the generated music, giving a more complex meaning to the piece. *Emotion* refers to the capability of the MGS to convey specific emotions with the generated music.

These two are treated together because it is possible to convey different emotions in different sections of the piece, one of the main aspects of *Narrative Adaptability*. Both of these can be seen as a special instance of *Control*, where the features that are being controlled relate to emotional aspects or to specific events of the narration. This is especially relevant in non-linear media (like video games) where the Narrative must adapt in real-time to the events in the media.

The study of Emotion in music has a long history (Juslin, 2010) and, as can be seen in the review by Williams et al. (2015), has often been considered in MGSs. Narrative Adaptability is less commonly found, despite the fact that such adaptability is something that human composers could never achieve without the help of a computer, making it an interesting field of investigation. Ventura et al. (2009) present an installation implementing a typical architecture for emotion-aware systems: an emotion (expressed as values in the valence/arousal plane; Hunter and Schellenberg, 2010) is detected (in this case by analysing the movements of the users via webcam) and then used as the input for the MGS. To do so, some features that are known to be related to emotional expression are manipulated, such as tempo, pitch range and loudness (Oliveira and Cardoso, 2007). A similar architecture is used by Scirea et al. (2018) to add music to Checkers: the MGS analyzes the board to understand how risky the situation is for the player, and then generates music that emotionally expresses the level of risk.

*Mezzo* by Brown D. (2012), Brown D. L. (2012) uses a different approach that takes its roots in classical music: the use of *Leitmotifs*. In a video game setting, some characters and situations are given a theme (composed by a human), and when those are encountered in the game, a message is sent to *Mezzo*. This will use the themes triggered by the messages, blending them together to generate a music that expresses the current situation. Similarly, when music is used within human-computer interaction, is useful to detect musical features in the human interaction to generate music that matches the emotional content as a feedback (Carnovalini and Rodà, 2019b; Carnovalini et al., 2019).

Another approach that does not necessarily involve a full MGS, but can be used to increase its adaptive capabilities, is that of automatically generating transitions between pre-composed sections, to be able to connect sections without knowing a priori when one will end and one will start (Horner and Goldberg, 1991; Gillespie and Bown, 2017). This is usually applied to human-made compositions, but it could be easily applied to a MGS, as long as it is capable of generating the next musical piece in advance, since it is needed for the transition generation.

These challenges are especially important for creativity, as they address “Intention & emotional involvement” and “Progression & development” and “Social interaction and communication,” and in general are fundamental for the affective perception of the machine, which in turn is important to pass Turing-like tests. One research direction we suggest is to study how different expressive features can influence each other, and how to select one specific expressive technique to convey certain feelings rather than altering all the features that are linked to that emotion, so that systems could be able to generate, for example, a sad piece which is also fast-paced. This can also improve “Variety, divergence & experimentation” of the generated pieces and possibly lead to more “Originality.”

### 4.3. Hybridization

*Hybridization* refers to the use of more than one technique for music generation in a single MGS.

This is the only point of this list that does not concern a quality of the output, but rather a characteristic of the system itself. The need to go beyond a single method for the generation of music was already noted 20 years ago (Papadopoulos and Wiggins, 1999), but the call for hybridization is relevant to this day. The rationale behind this idea is that since there is not a single method that has been proved to be more effective than the others, nor to be capable of addressing all the issues that a MGS must face, it is important to take advantage of different approaches. Nonetheless, using multiple algorithms is obviously expensive for the development, and in general it is hard to witness in the early stages of any project. Moreover, researchers are often more interested in applying a specific technique for music generation rather than creating a complete MGS that would benefit from hybridization.

Some approaches are more prone to being used in an hybrid context than others: we have already discussed how Genetic algorithms and rules or constraints are often coupled with other algorithms, but other approaches are possible. Eigenfeldt and Pasquier (2009) describes how the various versions of the *Kinetic Engine* have used different algorithms for designing agents capable of generating rhythm, melodies, and harmony. In the later versions, agents with different roles interacted with each other to generate both rhythm and melody, and also a Markov chain was used to influence the harmonic progression Eigenfeldt and Pasquier (2010). This gives an idea of how, in an agents-based system, it is possible to delegate different tasks to different agents, that can each implement a different strategy when generating their respective content.

A somewhat similar subdivision of tasks is proposed by Carnovalini and Rodà (2019a), where the process of composing a melodic phrase is divided in successive steps: generation of pitch succession, generation of rhythm, and finally generation of expressive variations of intensity and timing. Each of these steps follows a different algorithm, but some information is passed on through each step. In particular, all of the steps use information about the importance of each of the generated notes, dividing them with a Schenkerian approach (Simonetta et al., 2018). The authors argue that this idea can be further extended to other tasks (such as form generation and harmony), including both deep learning and classical AI algorithms, trying to find the optimal combination for each task (Carnovalini, 2019).

The use of expressive musical performance generation systems (Widmer and Goebl, 2004; Canazza et al., 2012, 2015), that are sometimes embedded in MGSs as the one just cited above, can also be seen as a form of hybridization, but will be better discussed in the next section.

We believe that systematic use of Hybridization could be one of the most prolific research direction for CC, since it could help researchers expand different dimensions of creativity using different techniques for each. Moreover, giving a variety of compositional approaches to a software could be seen as giving it a better “Domain competence,” and being able to choose between techniques can improve “Variety, divergence & experimentation.” Explicitly modeling into the software what different techniques are more apt for and changing behavior according to user's requests could be an interesting research direction.

### 4.4. Rendering

*Rendering* refers to the quality of the audio (meant as waveform) that is generated by the MGS.

This might seem redundant, as the quality of the generation is obviously important in a MGS. But in many cases, MGSs only handle symbolic music generation, usually as MIDI or MusicXML files, and the audio is generated with simple software MIDI synthesizers, which are far from giving good renditions of any musical composition.

We already mentioned that it is possible to add expressive performance to a generated piece in order to improve its audio rendering. This is usually done through existing algorithms that are applied to the music after it is generated. A review of existing algorithms can be found in Kirke and Miranda (2009).

Another way to improve the musical rendering is to use automatic orchestration techniques: rather than having a predetermined instrument to play the generated piece (piano seems to be a popular choice) it is possible to generate musical material that is then assigned to different instruments (Handelman et al., 2012) or to have a set of possible instruments from which to choose from and that can intervene at different moments of the composition (Anderson et al., 2013).

Brunner et al. (2018) describe a system that uses both expressivity and orchestration to perform Style Transfer through Variational Autoencoders (Kingma and Welling, 2013). Style Transfer tries to apply a certain style (for example, defined by a certain composer or genre) to an existing musical piece that was not originally meant for that style. This can of course be applied to computer-generated music as well, although we are not aware of any work in literature that has yet tried this approach.

All these approaches generate some sort of variations after the score generation process is over. This excludes any possibility to render audio in real-time, as the generation phase must be over for these algorithms to function. A different approach that has been less explored is to generate music and its expressive variation at the same moment: one example is PerformanceRNN (Oore et al., 2018).

A completely different approach is to model music directly at the audio level, thus implicitly generating the rendering as well. This approach is challenging for many reasons, including computational complexity and the difficulty to capture semantic structures from raw audio. Nonetheless, Dieleman et al. (2018) found that using Autoencoders it is possible to obtain realistic results that remain consistent for tens of seconds, meaning that local structures can be understood and modeled directly from the audio.

One could argue that the creative task we are interested in is the composition, while the rendering is delegated to musicians. While it is true that in some cases computers generate music that is meant to be played by humans, it is more often the case that computers directly play the generated music themselves. Moreover, in the context of evaluation of CC, having a good audio rendering can influence human evaluators, so it should not be overlooked (Oore et al., 2018; Carnovalini and Rodà, 2019a). More generally, Rendering can be seen as part of “Generating results”: while scores are results in themselves, the fruition of music is through sound. Therefore, to add “Value” to the output, Rendering must be considered. We are not aware of any research comparing user preference of computer generated music that is emotionally rendered vs. “deadpan” executions, but that would certainly be an useful contribution to CC research.

### 4.5. Structure and Mapping

*Structure* refers to generating longer pieces, containing reasonable repetitions and subdivision of different sections, usually recreating some kind of musical form. *Mapping* refers to the problem of handling different representations of music and choosing the most appropriate one for the generation of musical content.

While the first is notoriously difficult for MGSs, the latter is an issue that is often not considered, as usually a certain representation is chosen a priori. There are instead notable proposals in literature that further the possibilities for MGSs using specific representations of music.

Herremans and Chew (2016a, 2017) used a specific data structure, the *Spiral Array* (Chew, 2014), to compute the *tension profile* of a musical piece. This profile is used to generate a new piece that follows the same profile, through constraint programming. Starting from a specific representation for tension structures, the MGS is able to create longer pieces with convincing structure. One might argue that the structure is simply being copied, but a possible extension to this work could possibly generate novel tension patterns using the same ideas.

Representations based on Schenker's or on Lerdahl and Jackendoff's theories are studied, since these can capture different levels of structural information. Most works only have the aim of automatic analysis of musical pieces (Marsden, 2010; Marsden et al., 2013; Hamanaka et al., 2016, 2017), but others have used this approach to generate music that follows a defined structure (Groves, 2016; Carnovalini and Rodà, 2019a)

Other systems approached the problem of structure without any specific representation. GEDMAS (Anderson et al., 2013) explicitly generates structure, seen as successions of 8-bar segments, through a Markov chain. Medeot et al. (2018) describe StructureNet, a neural network that studies occurrences of repeats (either of rhythm or of interval sequences), and that can be embedded in a larger MGS influencing the generation process according to the learnt structures of repeats.

Structure is strongly linked with the creative dimension of “Progression & development,” and can be linked to the challenge of Narrative Adaptability as well. Once again, we suggest to hybridize different approaches, possibly using different techniques at every level of representation to consider the development of a piece at a macro level before considering the local melodic and harmonic content.

### 4.6. Playing Difficulty

*Playing Difficulty* refers to the ability of an MGS to regulate the difficulty for a human to play the generated music.

This can be seen as a specific instance of *Control*, where the feature that must be controlled is the technical difficulty of the output. This problem only becomes relevant when the output of the MGS is meant to be played by a human, which is often not the case: this might be the reason why this issue has hardly been acknowledged in literature.

The review by Herremans et al. (2017), that is the only one to mention Playing Difficulty, only cites a couple recent works that have considered the issue. One is Sabastien et al. (2012), that designed seven criteria used to estimate the difficulty of a piano piece, in order to suggest pieces to learn to students. A similar approach for guitar is presented in Xambó et al. (2018), based on known chords. Both these systems are not MGSs, but could be implemented as a constraint or as a fitness function in a MGS. Another work is that of McVicar et al. (2014), that generates guitar solos in tablature form. This is indeed a MGS, but it does not really consider the difficulty of the generated solo, but rather uses an algorithm to minimize fingering difficulty, without affecting the generation of the piece. Extending on the same idea, Ariga et al. (2017) created a guitar solo generator that considers the fingerings as a way to measure and control the difficulty of the generated solos. Nakamura and Yoshii (2018) describes a system that creates piano reduction of ensemble scores, capable of generating reductions with different levels of difficulty based on fingering and tempo information.

On the opposite side of the difficulty spectrum, Pachet (2012) describes a system that can generate virtuoso solos, using variable-order Markov chains trained on a dataset of virtuoso solos. Arguably, increasing the Playing Difficulty of a generated piece is easier than lowering it (without losing musicality), but the work by Pachet was motivated by a study of creativity in solos and not of difficulty itself.

To be able to change the playing difficulty of a piece, one needs to increase the “Domain competence” considering for example the physical characteristics of the instruments that will be used to perform the piece, making this challenge also relevant to the perception of CC. Choosing an appropriate level of difficulty can also improve the “Social interaction and communication,” since if one wishes to create a MGS to interact with humans, it is important to tune the difficulty to the end user's ability. One possibility to deepen this relatively unexplored branch is to use published exercises books for the learning of instruments to extract features correlated to the difficulty level.

## 5. Conclusions

We presented a broad introduction to the field of Computational Creativity, and to Music Generation Systems in particular. In the first part (section 2), we described the main concepts needed to understand research on creativity (both human and computational), analyzing a variety of definitions and studies on what makes People, Processes, Products be perceived as creative (to the Press). We reviewed some works on the evaluation of creativity, which is one of the main challenges for computational creativity, and is strongly intertwined with trying to give a formal definition of creativity. In the second part (section 3) we focused on the specific task of Music Generation, starting from existing reviews on the subject. We described the main approaches to Music Generation in the literature, giving examples for each. Finally (section 4), we listed a set of issues that need further development as identified by the reviews we analyzed. For each, we listed possible approaches used to face these issues that have been proposed by papers published in the last 10 years, discussing how facing these challenges can also lead to an improvement in the perceived creativity.

What hope that this review can serve as an introduction to the research on Music Generation that can give all the necessary bases to any researcher who wants to approach Music Generation from a Computational Creativity point of view. As we already underlined, the main problems in this field derive from the fact that often researchers fail to clearly state the goals of their research, and consequently cannot give a good evaluation of their work. This review can help frame new research within the scope of Computational Creativity, and give an indication of what still needs to be done. For instance, we believe that often researchers have chosen a specific method or algorithm and developed Music Generation Systems with the goal of using that method rather than trying to create a complete Music Generation System that could benefit from the use of different approaches to face different issues. To this goal, we advocate that a well studied hierarchical hybridization could give means to face many of the open challenges listed above, and possibly also allow for easier comparison between different methods, thus opening new possibilities for evaluation.

## Author Contributions

FC selected and studied the sources, designed the structure of the manuscript, and wrote the first draft of the manuscript. AR contributed with supervision over the study of the literature and the writing of the manuscript. All authors contributed to manuscript revision, read and approved the submitted version.

### Conflict of Interest

The authors declare that the research was conducted in the absence of any commercial or financial relationships that could be construed as a potential conflict of interest.

## Footnotes

^1^A web application developed by Wolfram Research, Inc. that allows users to generate music using Cellular Automata is available at http://tones.wolfram.com/generate/.
